# Usability, Relevance, and User Engagement of a Government-Sponsored and Community Co-Designed Digital Mental Health Website During the COVID-19 Pandemic: A Pilot Study on Latino/a/x Adults

**DOI:** 10.2196/72952

**Published:** 2026-06-10

**Authors:** Blanche Wright, Lily Zhang, Jocelyn I Meza, Lupita Rodriguez, Irisela Contreras, Vanessa Terán, Alexander S Young, Miriam Nuño, Tiffany Dzou, Sergio Aguilar-Gaxiola, Kenneth B Wells

**Affiliations:** 1 Department of Psychology, University of Oregon, 1451 Onyx Street, Eugene, OR, 97403-1227, United States, 541-346-4831; 2 Research Center for Health Services and Society, Jane and Terry Semel Institute for Neuroscience and Human Behavior, University of California, Los Angeles, CA, United States; 3 Department of Psychiatry and Biobehavioral Sciences, University of California, Los Angeles, CA, United States; 4 Health Education Council, Sacramento, CA, United States; 5 Mixteco Indígena Community Organizing Project, Oxnard, CA, United States; 6 Department of Public Health Sciences, University of California, Davis, CA, United States; 7 Smidt Heart Institute, Cedars-Sinai Medical Center, Los Angeles, CA, United States; 8 Department of Internal Medicine, University of California, Davis, CA, United States; 9 Center for Reducing Health Disparities, University of California, Davis, CA, United States; 10 Clinical and Translational Science Center, University of California, Davis, CA, United States; 11 Research Center for Health Services and Society, Jane and Terry Semel Institute for Neuroscience and Human Behavior, University of California, Los Angeles, Los Angeles, CA, United States; 12 Department of Health Policy and Management, University of California, Los Angeles, CA, United States

**Keywords:** Hispanic or Latino, digital health, COVID-19, community partnership, mental health, usability, relevance, engagement

## Abstract

**Background:**

Latino/a/x adults have higher rates of unmet mental health needs than other racial and ethnic groups. One promising solution to help bridge this gap in care is digital mental health tools. Digital tools, such as self-help websites, have demonstrated the ability to enhance mental health literacy, reduce stigma, and improve mental health symptoms. Despite the potential benefits, engagement remains a critical challenge, and there has been a large oversight of unique considerations for Latino/a/x adults as end users.

**Objective:**

Guided by the Technology Acceptance Model and the Behavioral Model for Vulnerable Populations, the study’s overarching objective is to characterize within-group variation of Latino/a/x adults’ engagement with a government-funded, prevention-focused mental health website that was co-designed with community partners during the COVID-19 pandemic.

**Methods:**

The Together for Wellness/Juntos Por Nuestro Bienestar (T4W/Juntos) website offered free digital mental health resources to help Californians cope with the COVID-19 pandemic. A pilot evaluation of the website involved baseline and 4-week follow-up surveys about demographics, behavioral health needs, and overall website user experience. This current subanalysis focused on a stratified sample of Latino/a/x adult participants (baseline N=131; baseline and follow-up n=68). The baseline sample was mostly female (106/130, 81.5%); 66.9% (87/130) preferred to use the website in English and 30% (39/130) preferred Spanish. Behavioral health needs were assessed using the Patient Health Questionnaire-2, Generalized Anxiety Disorder 2-item scale, and a COVID-19 stressors checklist. We measured usability, comfort using the website, relevance of the website, and past-month use of resources. Data were analyzed using ordered and standard logistic regression methods.

**Results:**

Latino/a/x adults who preferred using the website in English (odds ratio [OR] 13.76, *P*<.001) compared with Spanish were more comfortable using the website. Compared with adults aged 18‐30 years, adults aged 41‐50 years had significantly lower odds of agreeing that the website was easy to use. Sensitivity analyses revealed that participants who found the website easier to use (OR 2.22, *P*=.001) and those with greater behavioral health needs (OR 1.22, *P*=.045) were more likely to perceive the website’s topics as relevant. Participants with higher behavioral health needs at baseline were more likely to use the website and engage with resources for anxiety or stress at follow-up (OR 1.42, *P*=.047).

**Conclusions:**

This study addresses gaps in understanding Latino/a/x adults’ experiences with a prevention-focused mental health website. The language-based disparity in comfort highlights the need to significantly improve the user experience for Latino/a/x Spanish speakers. Still, the website can be a helpful resource for Latino/a/x adults with high behavioral health needs, bridging a critical gap in support. A collaborative approach to developing resource-rich websites with trusted community organizations is vital for effectively reaching Latino/a/x communities and tailoring resources to address their unique needs.

## Introduction

### Background

National surveys show that before and during the COVID-19 pandemic, Latino/a/x adults had higher unmet mental health needs than other racial and ethnic groups [1,2]. Language disparities exist such that English speakers are nearly twice as likely to engage in mental health care compared with Spanish-speaking adults [3]. Monolingual Spanish speakers and Latino/a/x adults with limited English proficiency are especially vulnerable to unmet mental health needs due to systemic barriers, such as the limited availability of Spanish-speaking providers. Indeed, as few as 4% of mental health service providers are linguistically competent to deliver services in Spanish [4]. In 2020, Latino/a/x adults’ mental health risk increased due to the disproportionate negative impacts from COVID-19 exposure and social determinants of health, such as housing and food instability [5,6]. The pandemic simultaneously exacerbated mental health problems and the ongoing national shortage of mental health professionals, thus creating an urgency for population-level interventions to better support Latino/a/x adults’ mental health [7]. Throughout this paper, we use the term Latino/a/x but acknowledge that Hispanic and Latiné are other terms used to describe individuals from Latin America.

Amidst the national mental health crisis, one promising solution is digital mental health tools (eg, websites, videos, smartphone apps, and online forums) [8]. These platforms offer accessible, potentially scalable options for addressing mental health needs, particularly in underserved populations. For instance, self-guided mental health websites have been found to increase mental health literacy, reduce stigma, and even improve mental health symptoms [8,9]. Despite the early promise of digital mental health solutions, research has largely overlooked Latino/a/x adults as end users [10]. This gap has resulted in a significant lack of literature on how Latino/a/x communities engage with digital tools, including mental health websites, leaving their specific needs and usage patterns underexplored. This oversight is particularly concerning, given the existing “digital divide,” which positions minoritized groups, such as Latino/a/x adults, as having limited access to technology, further impacting digital literacy [11]. Although digital equity has not yet been achieved, there has been progress—as of 2024, approximately 93% of Latino/a/x adults own smartphones [12]. Still, research supports access challenges such that Latino/a/x adults, especially Spanish speakers and those with low literacy or educational levels, are less likely to seek health resources online [13,14]. Their lower usage highlights a critical barrier to accessing digital health solutions for a group who may need them the most. Across all racial and ethnic groups, user engagement continues to be a major obstacle in maximizing the reach and impact of digital mental health tools. For example, a systematic review of mental health phone apps revealed a median 3.3% retention rate of users within a 30-day period [15]. To enhance engagement across diverse populations, experts recommend codeveloping digital mental health tools in collaboration with minoritized communities [14,16]. This approach is thought to ensure that the tools are both relevant and accessible to those they are intended to serve. Promoting the adoption of digital mental health tools among Latino/a/x consumers requires more research to explore how they engage with and evaluate digital resources, especially when resources are specifically tailored to address their cultural and linguistic preferences.

### Co-Design of the Together for Wellness/Juntos Por Nuestro Bienestar Website

One example of a digital mental health website that aligns with recommendations to co-design with traditionally underrepresented communities is the Together for Wellness/Juntos por Nuestro Bienestar (hereafter called T4W/Juntos) government-sponsored, prevention-focused mental health website [15,17]. T4W/Juntos launched in 2020, and was created to provide free digital mental health resources to help adults living in California navigate the challenges of the COVID-19 pandemic while promoting and maintaining their mental well-being. Using the World Health Organization’s Classification of Digital Health Interventions, T4W/Juntos would fall in the category of 1.6.1 “Digital Health Interventions for Persons, On demand communication with persons, Look-up of information on health and health services by individuals” [18]. The development of T4W/Juntos was grounded in community-partnered participatory research principles, such as embodying 2-way knowledge exchange and fostering trust between stakeholders [19]. The partnership brought together state governmental agencies, 11 community-based organizations across the state, researchers, technology experts, and advisory groups who collaboratively curated the website with resources that were stakeholder-informed, user-friendly, and tailored to meet the specific needs of underresourced communities. Many community partners represented and served Latino/a/x and Spanish-speaking populations, and the T4W/Juntos website was available in 13 languages (including English and Spanish). Both community partners and researchers vetted and reviewed free digital mental health resources available on other websites for six topics that the community identified as priority areas during the pandemic: (1) Learn About COVID-19 pandemic, (2) Soothe Anxiety and Stress, (3) Support Resilience of Kids and Families, (4) Cope With a Recent Loss, (5) Connect With People and Support Social Justice, and (6) Need to Talk to Someone (ie, helplines). Resources on the T4W/Juntos website included links to other websites (n=62), videos (n=18), YouTube links (n=2), PDF documents (n=32), apps (n=10), and helplines (n=9) [16]. Several resources were drawn from evidence-based interventions, such as mindfulness and cognitive behavioral therapy, while others were psychoeducational (eg, describing depression). Most resources were considered evidence-informed or evidence-based for specific groups rather than for universal prevention. As of 2025, the website continues to be updated based on community needs and partner feedback.

### T4W/Juntos-Integrated Framework and Initial Evaluation

An initial evaluation of T4W/Juntos was conducted using an integrated framework of the Technology Acceptance Model (TAM) and the Behavioral Model for Vulnerable Populations [18,20] to examine the engagement of 315 diverse adult participants and the potential of the website to improve anxiety and depression symptoms. This study also uses the integrated framework. The TAM is a leading model to examine the uptake of digital mental health tools, positing that “perceived usefulness” and “perceived ease of use” influence the actual use of digital tools [21]. The TAM acknowledges that “external variables” influence usability and uptake, but it does not specify which variables; moreover, due to inconsistent empirical findings, critics note that the TAM overlooks important factors [22]. One such factor is “comfort” using digital tools, as tools may be simple and easy to navigate but that does not necessarily indicate that a user will feel comfortable (ie, free from distress) when using the tool. “Comfort” was not mentioned explicitly in the original TAM article [21]; however, T4W/Juntos community partners suggested that the research team measure comfort in the initial evaluation. Existing studies using the TAM have conceptualized “comfort” as distinct from usability, positing that it influences engagement with technology [23,24]. In general, however, research examining comfort as a construct and its influence on the uptake of digital health and mental health tools are major gaps in the literature.

When considering the uptake of digital tools among Latino/a/x adults, the Behavioral Model for Vulnerable Populations can further address the TAM’s shortcomings. Specifically, the Behavioral Model outlines predisposing factors, such as age, language, and education, that influence health behavior (eg, use of a digital mental health tool), as well as enabling factors and barriers to uptake, which can then lead to improved health outcomes [25]. In the initial T4W/Juntos evaluation with 315 racial and ethnically diverse adult participants, Latino/a/x adults had a significantly higher engagement with the website than other racial and ethnic groups, and greater engagement was associated with lower depression symptoms [16]. While it is promising that Latino/a/x adult engagement was robust in the broader sample, they also reported higher discomfort using the website, suggesting that more support may be needed. Moreover, within-group variation of Latino/a/x adult engagement has not yet been examined for T4W/Juntos and in the broader literature. Examining these differences could offer valuable insights into which Latino/a/x subgroups benefit the most from the website and identify those who may require additional support to fully engage with and benefit from digital resources.

Two understudied but potentially influential factors on Latino/a/x adult engagement with digital tools are mental health needs (or “behavioral health needs” if including substance use) and perceived relevance of the tool. The Behavioral Model for Vulnerable Populations posits that perceived and evaluated health status (ie, “need”) positively influences engagement in health promoting behavior. A recent systematic review of barriers and facilitators on the use of digital mental health interventions described mixed results—some studies found that depressive symptoms spurred engagement with mental health websites, while other studies found that higher severity of depressive symptoms inhibited use [26]. The review also highlighted that engagement is facilitated when the digital mental health tool is perceived to be “relevant” [26]. Similar to behavioral health needs, relevance is often considered an essential precursor to engagement with digital mental health tools [22,23,25]. Although relevance is not explicitly represented in the TAM, a few studies have shown that relevance positively influences the TAM elements, “usefulness” and “behavioral intention to use” digital tools [24,26,27]. Further exploration of relevance within the context of need and usability can clarify factors most central to fostering user engagement. In applying the Behavioral Model of Vulnerable Populations, comfort along with usability can be captured as enabling factors, or barriers to care depending on whether they are high (ie, enabling) or low (ie, a barrier). In general, there is limited research characterizing the extent to which factors, such as usability, comfort, and relevance, influence the engagement of Latino/a/x adult users with digital mental health tools. For instance, while lower education has been linked to lower engagement with digital mental health tools [26], little is known about its relationship with usability and in Latino/a/x samples.

### Study Aims

**Figure 1.**
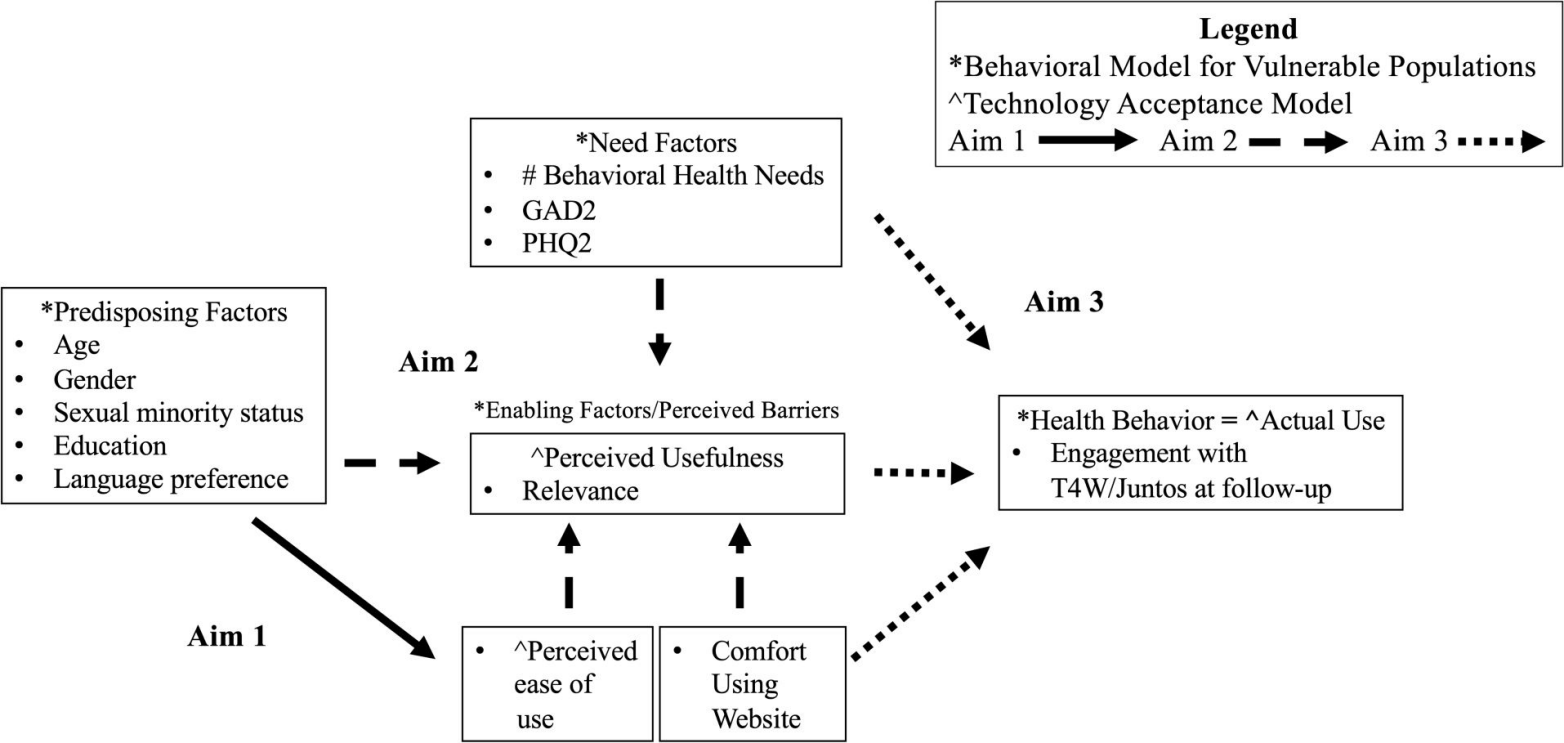
Integrated conceptual framework of study aims based on elements from the Technology Acceptance Model and the Behavioral Model for Vulnerable Populations. GAD2: Generalized Anxiety Disorder 2-item scale; PHQ-2: Patient Health Questionnaire-2.

This study is a subanalysis of the broader T4W/Juntos evaluation and focuses on characterizing within-group variation of Latino/a/x adults’ uptake of T4W/Juntos; results will act as a pilot for future research. As depicted in Figure 1, the 3 aims of the pilot study are guided by an integrated conceptual framework based on the TAM and Behavioral Model of Vulnerable Populations. First, we sought to identify the demographic factors that were associated with website ease of use, given that poor usability may act as a barrier to engagement. We also explored the relationship between demographic factors and comfort as it has not been a major focus in the digital health literature. We hypothesized that Latino/a/x adults with higher levels of education and a preference for the English website would be more likely to rate the website’s ease of use and comfort more favorably. Aim 2 examines the extent to which demographic and psychosocial characteristics and behavioral health needs during the pandemic influence perceived relevance of website topics. Understanding these factors can shed light on what drives Latino/a/x adults to find the content meaningful and engaging. Although aim 2 was primarily exploratory, we anticipated that individuals with greater behavioral health needs would perceive the website’s content as more relevant. The third objective was to assess whether behavioral health needs, perceived relevance of the content, website’s ease of use, and comfort were positively associated with uptake, measured by use of resources within the past month. Aligned with the Behavioral Model, we anticipated that Latino/a/x adults with greater behavioral health needs would be more likely to actively engage with the website’s resources, using them at higher rates. The findings from this pilot evaluation can provide valuable insight into the factors that drive Latino/a/x adults’ engagement with digital mental health prevention tools. As the website was created for users in California, there is no current plan to expand nationally. These insights can inform strategies to reduce racial and ethnic disparities in unmet mental health needs, helping bridge gaps in access and care for Latino/a/x communities.

## Methods

### Study Context and Procedures

T4W/Juntos website development was initiated by the California Health Care Services Division of Behavioral Health and the California Mental Health Services Oversight and Accountability Commission using funding from the Federal Emergency Management Agency and the Substance Abuse and Mental Health Services Administration. More detailed accounts of the development of T4W/Juntos and resources are described elsewhere [20]. The T4W/Juntos website that was used for the initial evaluation has shared ownership between Chorus Innovations Inc. and academic collaborators. This subanalysis uses data from the initial evaluation of the T4W/Juntos website. The evaluation was developed in partnership with the 11 community-based organization partners who signed an agreement and were compensated with an initial payment of US $1000. Community partners provided input on survey measures and design.

Eligibility criteria were that participants (1) had internet access, (2) could speak English or Spanish, and (3) were aged 18 years or older. Partners were asked to invite clients, partners, and staff to participate in the evaluation through their email listserv. For agencies with large lists of potential participants, random selection tools were provided. Assuming a 50% response rate, we encouraged the agencies to invite 80‐100 individuals to ultimately enroll 30‐40 per agency; that number was later expanded to 70 per agency to increase enrollment. For the evaluation, we created a duplicate version of the entire public access T4W/Juntos website that allowed us to track enrollment by the inviting agency. Potential participants were emailed invitations (with 1‐2 reminders) and a link to the eligibility screener. Consented participants were given a link to the duplicate website and asked to complete a baseline survey after visiting the website.

On the baseline survey, participants were asked about their perceptions of the website, mental health status, and service utilization. Of the participants, 9.2% (29/315) of the survey participants of the full evaluation were invited to do a qualitative phone interview 2 weeks after the baseline survey. Qualitative approach, protocol, and results are described elsewhere, and qualitative data are not used in this study [28]. All participants were invited to complete a follow-up survey 4‐6 weeks after their baseline. Surveys were conducted between September 20, 2021, and April 4, 2022, for baseline, and between October 22, 2021, and May 17, 2022, for the follow-up. The current subanalysis had 1 stratification eligibility criterion: the participants indicated that their race and ethnicity was Latino/a/x.

### Ethical Considerations

Evaluation of the initial evaluation of the T4W/Juntos website was approved by the University of California Los Angeles Institutional Review Board for Human Subjects (20‐002163-AM-00008). Participants who were invited to participate were provided with an online link to the web-based consent form and clicked to give consent. All participants provided informed consent online and were advised that participation was voluntary, data would be deidentified, their contact information would be used for follow-up, and linking data (ie, using an ID code) would be stored on a secure server separately from study data. Informed consent was affirmed at follow-up assessments. Inviting agencies were not informed of the participation of individuals. The study team used the separately stored contact information to invite participants to follow up. Participants received a US $25 electronic gift card for each survey and interview they completed. Participants did not have access to data but could skip questions. Finally, we used the iCHECK-DH Guidelines and Checklist for the Reporting on Digital Health Implementations to ensure completeness of reporting of this study.

### Measures

#### Demographic Characteristics

Participants self-reported their age, gender identity, race and ethnicity, sexual orientation, and highest level of education on the baseline survey. They were also asked what language they preferred to use the website in. For educational level, we created a categorical variable: less than high school or high-school graduate, some college, college graduate, and graduate school.

#### COVID-19 Stressors

At baseline, we measured the number of COVID-19 stressors with 18 items from the Pandemic Stress Index [29]. Eight items assessed behavioral health-related difficulties experienced during the pandemic: worrying about friends, family, or partners, and so forth; more anxiety; more depression; more or less sleep or other changes to normal sleep pattern (hereafter referred to as “sleep changes”); increased alcohol or substance use; loneliness; frustration or boredom; and getting emotional or social support from family, friends, partners, a counselor, or someone else. Ten items primarily captured infection-related and resource-related stressors: diagnosed with COVID-19 disease; fear of getting COVID-19 disease; fear of giving COVID-19 disease to someone else; confusion about COVID-19 disease and prevention; stigma or discrimination from other people; personal financial loss; not having enough basic supplies (eg, food, water, medications, and a place to stay); getting financial support from family, friends, partners, an organization, or someone else; change in sexual activity; and other difficulties or challenges. A count variable of all 18 items represented the full list of stressors experienced during the COVID-19 pandemic such that a higher number indicated more stress.

#### Behavioral Health Needs

Behavioral health needs were operationalized with 3 measures collected at baseline. From the Pandemic Stress Index items, we used the 8 items that captured behavioral health needs to create the count variable “COVID Stressors: Behavioral Health,” which indicates higher behavioral health needs during the pandemic. The second measure of behavioral health needs was the Patient Health Questionnaire-2 (PHQ-2), which assessed baseline depression symptoms [30], and the third was the Generalized Anxiety Disorder 2-item scale (GAD-2) to measure baseline anxiety symptoms [31]. The PHQ-2 has 2 items that assess depressed mood and anhedonia, and the GAD-2 has 2 items that assess feeling nervous or anxious and excessive worrying. Both are measured on a frequency scale for the past 2 weeks (0 = not at all; 3 = nearly every day), with scores of ≥3 indicative of moderate depression or anxiety. The PHQ-2 and the GAD-2 have been found to be valid measures for Latino/a/x and Spanish-speaking adults [28,32].

#### Website Ease of Use, Comfort, and Relevance

At baseline, participants were asked to rate their level of agreement on a 5-point Likert agreement scale (Strongly Disagree to Strongly Agree; plus don’t know) with the state “The website is easy to use,” which was adapted from the System Usability Scale [33]. To assess comfort, participants used the same 5-point Likert scale in response to the statement, “I do not feel comfortable using this website”; comfort was reverse scored in analyses. Relevance of topics on the website was also assessed at baseline using the same 5-point Likert agreement scale to the item, “The topics covered on the website were relevant to me.” The research team developed the comfort and relevance statements based on the TAM [21], extant literature [34-37], and input from the community partners. In the previous main evaluation of T4W/Juntos, comfort was used as a single item; ease of use and relevance were used in an aggregate score with satisfaction (Cronbach α=0.74) [16]. In the current subanalysis, we used each item as a single measure in an effort to more granularly characterize Latino/a/x adult user perspectives on digital resources. Single-item measures are considered efficient and are valid measures when the construct is unambiguous like the main constructs used in this study [38].

#### Past Month Engagement at Follow-Up

The follow-up survey asked questions about participants’ engagement with the website during the past month. We used yes/no questions asking whether they used the website, and which of the six categories of resources they used during the past month period: (1) Learn About COVID-19, (2) Soothe Anxiety and Stress, (3) Support Resilience of Kids and Families, (4) Cope With a Recent Loss, (5) Connect With People and Support Social Justice, and (6) Need to Talk to Someone? As used in the initial T4W/Juntos evaluation [16], total previous month use of resources was computed by summing the yes/no questions about the 6 categories used; a higher score indicated more use or engagement with the website.

### Statistical Analysis

All analyses were conducted in Stata (version 15.1; StataCorp LLC) [39]. To obtain sample characteristics, we ran basic descriptives to report the mean and SDs for continuous variables, and frequencies for categorical variables. To compare the baseline and follow-up samples, we conducted 2-tailed *t* tests and chi-square tests to identify significant differences. For aim 1 analyses, we used 2 ordered logistic regression models to assess predictors of ease of use and comfort with the website, with age, educational level, language preference, gender, and sexual minority status as predictors.

For aim 2, we used ordered logistic regression models to examine predictors of the perceived relevance of the website’s topics. Key predictors included COVID stressors, behavioral health needs, ease of use, comfort, age, language, gender, and sexual minority status. These analyses aimed to uncover which factors were most strongly influenced by participants’ perceptions of the site’s relevance to their experiences. We sought to examine whether behavioral needs specifically influenced perceived relevance, independently of general stress levels during the pandemic; thus, we first ran a main model incorporating the full range of COVID stressors and then ran a sensitivity analysis focused only on the subset of stressors related to behavioral health. This analysis allowed us to isolate the unique influence of behavioral health needs on perceived relevance. Given that the count of the “COVID Stressors: Behavioral Health” included 1 item assessing “more anxiety” and 1 item assessing “more depression,” we investigated potential issues of multicollinearity with PHQ-2 and GAD-2 in the model by assessing Variance Inflation Factors. In both the main model and sensitivity analysis, Variance Inflation Factors scores fell within acceptable established thresholds [40] indicating no significant multicollinearity issues. As a result, we retained PHQ-2 and GAD-2 scores in the models to ensure a comprehensive assessment of behavioral needs.

Finally, for aim 3, we conducted tabulations to report the proportion of adults who indicated that they used the website within the past month. We also provided a detailed breakdown of the proportion of participants who engaged with each of the 6 resource categories available on the website during that period. To determine whether there were significant differences between adults who visited the website and those who did not, we conducted 2-tailed *t* tests and chi-square analyses on sociodemographic and behavioral health need variables. We performed logistic regression analyses to identify predictors of visiting the website within the past month, as well as the likelihood of using each of the 6 resource categories in the past month. We analyzed specific resource categories to determine which types of digital content Latino/a/x adults with behavioral health needs were more likely to engage with. This focused examination allowed us to identify resources that may be the most relevant and appealing for this population. We also performed a linear regression analysis to identify predictors of the total number (out of 6) of resource categories accessed by participants. Considering the modest follow-up sample size (n=68), we applied a parsimonious approach in selecting independent variables, ensuring that only the most relevant predictors were included in the analysis to maintain statistical power and avoid overfitting. The independent variables included the number of COVID-19–related behavioral health stressors, perceived relevance of the website, and comfort using the website. The number of COVID-19–related behavioral health stressors was chosen as a comprehensive measure of need, instead of separately entering GAD-2 and PHQ-2 scores. Age, education, and language preference were included as covariates to account for demographic influences on engagement.

## Results

### Participant Characteristics

Table 1 provides details about demographic characteristics, behavioral health need levels, and pandemic stressors. The baseline sample included 131 Latino/a/x adults, who were primarily female (106/131, 81.5%) and had an average age of 37.84 (SD 12.13; range 18‐67) years. The follow-up sample consisted of 68 Latino/a/x adults, who were also mostly female (55/68, 82.1%), with a mean age of 38.13 (SD 12.15) years. The average number of COVID Stressors: Behavioral Health type in the baseline sample was 3.06 (SD 2.23; range 0‐8), and 3.18 (SD 2.07; range 0‐7) in the follow-up sample. Chi-square analyses and 2-tailed *t* tests revealed no significant differences between participants who completed the follow-up and those who did not. Although the participants represent only a small fraction of the total Latino/a/x population living in California, mapping of the reported zip codes of our participants showed that we had representation from Northern, Central, and Southern California.

**Table 1.** Demographics and baseline behavioral health need scores of Latino/a/x adults who participated in baseline and follow-up surveys, California, United States (2021‐2022).

Characteristics	Baseline sample	Follow-up sample
Total, n	131	68
Age (years), responses, n, mean (SD)	128, 37.84 (12.13)	67, 38.13 (12.15)
Sex, n (%)		
Responses, n	130	67
Female	106 (81.5)	55 (82.1)
Male	21 (16.2)	12 (17.9)
Other	3 (2.3)	0
Sexual minority, n (%)		
Responses, n	125	64
Yes	21 (16.8)	7 (10.9)
No	104 (83.2)	57 (89.1)
Education, n (%)		
Responses, n	131	68
Less than high school or high-school graduate	31 (23.7)	15 (22.1)
Some college	40 (30.5)	23 (33.8)
College graduate	49 (37.4)	25 (36.8)
Graduate school	11 (8.4)	5 (7.4)
Language preference, n (%)		
Responses, n	130	67
English	87 (66.9)	41 (61.2)
Spanish	39 (30.0)	24 (35.8)
Other	4 (3.1)	2 (3.0)
COVID stressors: full list^a^, n, mean (SD)	131, 5.70 (3.88)	68, 6.18 (3.77)
Baseline behavioral health needs		
COVID Stressors: Behavioral Health^b^, n, mean (SD)	131, 3.06 (2.23)	68, 3.18 (2.07)
PHQ-2^c^, responses, n, mean (SD)	131, 1.36 (1.51)	68, 1.49 (1.50)
GAD-2^d^, responses, n, mean (SD)	131, 1.53 (1.66)	68, 1.62 (1.78)

a Count variable of full list of dichotomous variables asking about COVID-19 stressors including behavioral health needs.

b Count variable of select dichotomous variables asking about behavioral health needs during COVID-19 pandemic: worry about friends, family, or partners, and so forth; more anxiety, more depression, sleep changes, increased alcohol or substance use, loneliness, frustration or boredom, and getting emotional or social support.

c PHQ-2: Patient Health Questionnaire-2.

d GAD-2: Generalized Anxiety Disorder 2-item scale.

### Aim 1: Baseline Website Ease of Use and Comfort

Models examining predisposing factors (ie, demographic factors) as predictors of website usability are summarized in Table 2. Language was significantly associated with comfort using the website. Latino/a/x adults who preferred the English version of the website had significantly higher odds—13.76 times greater—of feeling comfortable navigating the website than those who preferred the Spanish version (odds ratio [OR] 13.76, 95% CI 5.22‐36.28; *P*<.001). No other demographic factors were associated with comfort using the website. Age and educational level were significantly associated with website ease of use. Compared with adults aged 18‐30 years, adults aged 41‐50 years had significantly lower odds of agreeing that the website was easy to use (OR 0.32, 95% CI 0.11-0.90; *P*=.03). In contrast, the odds of agreeing that the website was easy to use was 4.04 times higher for adults who had at least attended some college than for adults who had a high-school education or below (95% CI 1.41‐11.60; *P*=.03). Language (ie, Spanish or English), gender, and sexual minority status were not significantly related to website ease of use.

**Table 2.** Predisposing demographic factors’ associations with comfort using website and perceived ease of use of Latino/a/x adults who completed the baseline survey, California, United States (2021‐2022).

	Comfort using website (N=131)			Website ease of use (N=131)		
	OR^a^	95% CI	*P* value	OR	95% CI	*P* value
Age (reference 18‐30), years						
31‐40	0.95	0.38‐2.36	.91	0.44	0.17‐1.17	.10
41‐50	0.62	0.24‐1.57	.31	0.32	0.11 - 0.90	.03^b^
50+	0.43	0.15‐1.25	.12	1.20	0.41‐3.53	.74
Education level (reference: less than high school or high-school graduate)						
Some college	2.51	0.97‐6.51	.06	4.04	1.41‐11.60	.01^b^
College graduate	1.92	0.79‐4.66	.15	2.44	0.95‐6.26	.06
Graduate school	1.05	0.27‐4.05	.94	2.34	0.60‐9.08	.22
Language preference (reference: Spanish)						
English	13.76	5.22‐36.27	<.001^c^	0.99	0.41‐2.35	.99
Other	5.29	0.67‐42.00	.12	0.24	0.03‐1.79	.17
Sex (reference: female)						
Male	0.56	0.22‐1.41	.22	0.47	0.17‐1.25	.13
Other	1.47	0.11‐19.20	.77	3.70	0.27‐51.32	.33
Sexual minority (reference: no)						
Yes	1.69	0.60‐4.78	.32	0.47	0.17‐1.27	.14

a OR: odds ratio.

b *P*<.05.

c *P*<.001.

### Aim 2: Baseline Website Relevance

Table 3 summarizes results from the main model and sensitivity analysis predicting perceived relevance of website topics measured at baseline. In the main model, we found that the full list of COVID stressors was positively associated with relevance; when replaced with COVID stressors of the behavioral health type in the sensitivity analysis, for each additional behavioral health stressor, the odds of agreeing that the website topics were relevant significantly increased by 1.22 (OR 1.22, 95% CI 1.00‐1.47; *P*=.045). In both the main and sensitivity analyses, we found ease of use to be positively associated with increased odds of agreeing that the website topics were relevant. In the main model but not sensitivity analysis, age was significant such that adults aged 31‐40 years had lower odds of agreeing that the website was relevant than adults aged 18‐30 years (OR 0.36, 95% CI 0.13-0.99; *P*=.048). Comfort using the website, PHQ-2, GAD-2, language, and sexual minority status were not significantly associated with relevance. Although the “other” gender was positively associated with relevance in the main model, interpretation is limited due to the small sample size of 3 participants in the “other” gender category.

**Table 3.** Associations of predisposing demographic factors, need, ease of use, and comfort with perceived relevance completed at baseline survey by Latino/a/x adults, California, United States (2021‐2022).

	Baseline—relevance of website topics (n=131)					
	Main model			Sensitivity analysis^a^		
	OR^b^	95% CI	*P* value	OR	95% CI	*P* value
COVID Stressors: Full List^c^	1.13	1.01‐1.27	.04^d^	—^e^	—^e^	—^e^
Baseline Behavioral Health Need						
COVID Stressors: Behavioral Health^f^	—^e^	—^e^	—^e^	1.22	1.00‐1.47	.045^d^
PHQ-2^g^	0.87	0.62‐1.24	.45	0.88	0.62‐1.25	.48
GAD-2^h^	1.08	0.75‐1.56	.67	1.08	0.76‐1.55	.66
Comfort using website	0.82	0.59‐1.14	.25	0.83	0.60‐1.15	.26
Website ease of use	2.15	1.38‐3.37	.001^d^	2.22	1.41‐3.49	.001^d^
Age (reference: 18‐30), years						
31‐40	0.36	0.13 - 0.99	.048^d^	0.36	0.13‐1.01	.05
41‐50	0.79	0.24‐2.56	.70	0.86	0.27‐2.74	.79
50+	0.95	0.29‐3.09	.93	0.92	0.29‐2.96	.89
Language preference (reference: Spanish)						
English	0.77	0.26‐2.28	.64	0.74	0.25‐2.18	.59
Other	1.18	0.16‐8.96	.87	1.11	0.15‐8.45	.92
Sex (reference: female)						
Male	0.87	0.32‐2.37	.79	0.95	0.35‐2.54	.91
Other	14.92	1.03‐216.04	.048^d^	13.62	0.95‐195.84	.06
Sexual minority (reference: no)						
Yes	0.53	0.19‐1.52	.24	0.58	0.21‐1.64	.30

a Sensitivity analysis uses COVID-19 Stressors in the Behavioral Health category only.

b OR: odds ratio.

c Count variable of full list of dichotomous variables asking about COVID-19 stressors including behavioral health needs: diagnosed with COVID-19 disease; fear of getting COVID-19 disease; fear of giving COVID-19 disease to someone else; confusion about COVID-19 disease and prevention; stigma or discrimination from other people; personal financial loss; not having enough basic supplies (eg, food, water, medications, and a place to stay); getting financial support from family, friends, partners, an organization, or someone else; change in sexual activity; other difficulties or challenges; worry about friends, family, or partners, and so forth; more anxiety; more depression; sleep changes; increased alcohol or substance use; loneliness; frustration or boredom; and getting emotional or social support.

d *P*<.05.

e Not available.

f Count variable of select dichotomous variables asking about behavioral health needs during COVID-19 pandemic: worry about friends, family, or partners, and so forth; more anxiety; more depression; sleep changes; increased alcohol or substance use; loneliness; frustration or boredom; and getting emotional or social support.

g PHQ2: Patient Health Questionnaire-2.

h GAD2: Generalized Anxiety Disorder 2-item scale*.*

### Aim 3: Past Month Engagement With Website Resources Measured at Follow-Up

Table 4 reports on the proportion of adults who visited the website during the previous month, and the proportion of adults who used each of the 6 categories. At follow-up, 67.8% (46/68) of the participants reported visiting the website or used resources downloaded from the website at least once. *T* tests and chi-square tests revealed no significant differences in demographics and need variables between participants who visited the website in the past month and those who did not. However, compared with those who did not visit the website, trends in the data revealed that a higher proportion of T4W/Juntos past month site visitors were Spanish speakers (16/40, 40.0%) and less educated (25/41, 60.9% did not complete college). The average number of resources used during the previous month was 2.99 (SD 2.42; range 0‐6). The most commonly used resource category was Soothe Anxiety and Stress, which had the exact same proportion of adults who visited the website in the past month (ie, 46/68, 67.8%). The least common resource category used was Cope With a Recent Loss (25/67, 37.3%).

**Table 4.** Past month engagement with website and resource categories reported by Latino/a/x adults at the follow-up survey, California, United States (2021‐2022).

	Follow-up sample (n=68)	
	N (% Yes)	
Visited website or used resources during past month	46 (67.75)	
Resource categories used		
Soothe Anxiety and Stress	46 (67.75)	
Learn About COVID-19	39 (58.21)	
Connect With People and Support Social Justice	35 (51.47)	
Support Resilience of Kids and Families	32 (47.06)	
Need to Talk to Someone (ie, helplines)	26 (38.24)	
Cope With a Recent Loss	25 (37.31)	

Table 5 summarizes the significant results from the ordered logistic regressions. When predicting past month use, and use of Soothe Anxiety and Stress, we found the exact same model results in ORs, CIs, and *P* values; the only significant predictor was COVID Stressors: Behavioral Health, such that for each additional behavioral health stressor, the odds of visiting T4W/Juntos and using Soothe Anxiety and Stress resources increased by 1.42 (OR 1.42, 95% CI 1.01‐2.01; *P*=.047). Upon a post hoc investigation of a cross-tabulation between past month use and use of Soothe Anxiety and Stress, we found that every person who used the website in the past month also used Soothe Anxiety and Stress resources; effectively, the binary outcomes were the same, hence why results were exactly the same.

For Talk to Someone, higher perceived relevance (OR 0.40, 95% CI 0.17-0.95; *P*=.04) and higher comfort using the website (OR 0.53, 95% CI 0.30-0.94; *P*=.03) predicted lower odds of engaging with the Talk to Someone resource. There were no significant associations when predicting the sum of the resource categories used and specific use of the following resource categories: Learn About COVID-19, Support Resilience of Kids and Families, Cope With a Recent Loss, and Connect With People and Support Social Justice. The covariates, age, education, and language, had no significant association in any of the models except that those aged 41‐50 years had lower odds of using the Talk to Someone resource category than those aged 18‐30 years (OR 0.10, 95% CI 0.01-0.93; *P*=.04).

**Table 5.** Baseline behavioral health need, usability, and comfort associations with past month engagement with website resources measured at follow-up with Latino/a/x adults, California, United States (2021‐2022)^a^.

	Follow up—engagement with resources (n=68)					
	Past Month Use and Soothe Anxiety and Stress			Talk to Someone		
	OR^b^	95% CI	*P* value	OR	95% CI	*P* value
COVID Stressors: Behavioral Health^c^	1.42	1.01‐2.01	.047^d^	1.09	0.84‐1.86	.56
Relevance of Website Topics	0.85	0.38‐1.88	.69	0.40	0.17-0.95	.04^d^
Comfort using website	0.81	0.47‐1.39	.44	0.53	0.30-0.94	.03^d^

a Covariates: age, education level, and language preference.

b OR: odds ratio.

c Count variable of select dichotomous variables asking about behavioral health needs during COVID-19 pandemic: worry about friends, family, or partners, and so forth; more anxiety; more depression; sleep changes; increased alcohol or substance use; loneliness; frustration or boredom; and getting emotional or social support.

d *P*<.05.

## Discussion

### Principal Results

The current subanalysis characterizes Latino/a/x adults’ perceived usability, comfort, and actual uptake of free digital mental health resources featured on a government-sponsored website, which was developed in a community-academic partnership to support the well-being of adults living in California during the COVID-19 pandemic. Analyses were devised from an integrated conceptual framework based on the Behavioral Model for Vulnerable Populations and TAM [20], with the explicit addition and exploration of comfort. Although most studies have linked higher education and English-language preference to greater engagement (ie, actual use) with digital mental health tools [11,12,21], our study adds exploration of these factors as determinants of usability, relevance, and comfort using a mental health website. Our findings partially support older age (ie, 41‐50 years vs 18‐30 years) and higher education as predisposing factors for higher perceived ease of use within the integrated model. For education, adults with at least some college versus those at or below a high-school education found it easier to use the website; importantly, there was also a positive trend for college graduates. Prior to the pandemic, collegiate education had already relied heavily on the internet or technology [41]; adults with at least some college experience likely had prior practice exploring websites, which may have made it easier to navigate the T4W/Juntos website. As the pandemic galvanized technology integration into high-school education, usability research may find diminishing education-based differences between high-school graduates and college students in studies conducted since March 2020, when the COVID-19 pandemic was declared a public health emergency. One gap that persists is the extent to which education below high school (eg, completing elementary school only) influences perspectives about and use of digital mental health resources; this is an area that deserves more attention in, as an estimated 1 in 4 migrants has less than a high-school education [42].

To further assess within-group variation, we measured language-based differences, finding that comfort using the site was rated higher by Latino/a/x adults who preferred the English website than by adults who preferred Spanish. There may be several reasons for this pattern, including that more online health information is available in English and thus more accessible to English-dominant speakers [1,43]. Moreover, Latino/a/x adults who use Spanish as their primary language have lower odds of looking to the internet for health or mental health support [11,12]. Studies also show that non–English-speaking adults have difficulties with digital literacy, which is the ability to find and use technology-based health resources [33-35]. Still, technology interaction experts posit that promoting usability must go beyond language translation, and digital tool developers should consider cultural context in their design [44]. Indeed, translating digital tools from English to Spanish is considered a surface structure cultural adaptation as it aims to fit the preferred language of users; deep structure cultural adaptations aim to increase receptivity and acceptance by incorporating cultural, social, and historical influences on health behavior [45]. While translation is a surface-level adaptation, preference for the Spanish language has been considered a proxy for lower acculturation to the US context or greater enculturation to heritage cultural values [46]. It may be that Latino/a/x adults who are less acculturated to the United States (ie, prefer Spanish) are less familiar with the availability of online resources. They may also align with the cultural value of *personalismo,* which includes friendliness and “making an interpersonal connection” when using health supports [47]. Alignment with *personalismo* may manifest as Latino/a/x Spanish–speaking adults preferring to speak to someone about their mental health needs rather than reading about them. Mental health website curators may consider ways in which to incorporate personal connection in design, resources offered, and the way in which resources are shared (eg, having videos of Latino/a/x adults sharing mental health information).

Through the lens of cultural sensitivity for Latino/a/x users, human support during website usage may be especially valuable. Human assistance may be provided by “technology-savvy family members” or video-based training, both of which have been found to increase digital literacy [48]. Although the field lacks consensus about which dimensions of culture influence usability, qualitative methods are recommended to evaluate cross-cultural usability [49], and this work has been published elsewhere for T4W/Juntos [28]. Understanding the role of cultural values in user engagement, including the level of psychological comfort using digital tools, can demystify why particular cultural groups may be more willing to engage in specific mental health content or digital modalities, and thus clarify which cultural considerations are worth investing in within the digital mental health space [50]. More research is needed to test the hypothesis that high *personalismo* values would indicate a need for human support.

Our study also contributes to the literature by assessing determinants of comfort and relevance, which are currently understudied—most research has primarily focused on usability, the links between ease of use and perceived relevance of digital mental health content on increased engagement [24,27,41]. Although gender and age did not remain significant determinants in sensitivity analyses, ease of use did. Perceived ease of use was associated with increased odds of reported relevance of website topics, which underscores the importance of developing strategies to promote usability among Latino/a/x adults. Aligned with our hypothesis, as the number of behavioral health needs increased during the pandemic, Latino/a/x adults perceived the mental health website as more relevant. This promising result may speak to the codevelopment and design process of T4W/Juntos, as several community partners prioritized Latino/a/x populations when selecting and vetting digital resources. When examining engagement at follow-up, as the number of behavioral health needs increased, the odds of engaging with anxiety or stress resources also increased. Overall, anxiety or stress was the most used topic. This makes sense as the pandemic spurred increased stress and worries in the general population, but Latino/a/x adults had disproportionately higher risk [49]. Our findings help validate the application of the Behavioral Model for Vulnerable Populations in digital mental health since higher behavioral health need predicted the health-promoting behavior of website usage.

Interestingly, relevance and comfort using the website were not associated with higher odds of overall past month engagement or use of specific resources. Moreover, the lack of association between relevance and past month use of website resources is contradictory to previous literature finding a positive relationship between relevance and uptake [24,27,41]. This may be due to proposed mechanisms in the TAM that we were unable to measure, including “attitude toward using” and “behavioral intention to use” [21]. Latino/a/x adults may also encounter logistical barriers to using the website that we did not measure, such as limited time and fatigue from using the internet at a high rate during the pandemic, or attitudinal barriers such as stigma that could lead to avoidance. Future research would benefit from both comprehensively measuring TAM constructs and assessing barriers to engagement among Latino/a/x adults.

Finally, higher ratings of relevance and comfort predicted lower odds of using the website link to talk to someone via crisis lines. It is possible that being comfortable using a website that seems relevant can facilitate the application of resources into one’s life, and thus prevent the need for more intensive person-delivered clinical support, which is costly. However, such claims must be empirically tested, as it is also possible that individuals did not opt to talk to someone for other reasons, including stigma-related worry about whether calls are anonymous, anxiety-related avoidance, or simply not being in acute distress that warranted help or hotline support. Moreover, availability of helplines and hotlines is unavailable in indigenous languages, which poses a barrier for Mesoamerican communities. In addition, findings raise additional questions about client decision-making when both human and digital resources are available, as clients have more direct power in accessing and using digital tools but person-delivered care may be more appropriate for certain clinical needs [51]. Regarding coordination of care, current research shows that health organizations must grapple with several factors, including readiness for change and the clinical appropriateness of digital supports for clients, for successful integration of digital tools [52]. For Latino/a/x adults, there may also be a preference for human interaction for health care needs due to *personalismo,* which could be a direction for future digital mental health research. Still, with the national mental health workforce shortage paired with language being a well-documented barrier to care, digital mental health resources and tools have the potential to fill an important care gap for Latino/a/x communities.

### Limitations

Although the subanalysis makes valuable contributions to the literature, findings should be considered within several limitations. First, data were not collected on some elements of the TAM (eg, attitudes toward using, behavioral intention to use, and usefulness) and the Behavioral Model for Vulnerable Populations (eg, enabling factors such as personal or family resources), which limited our ability to test a fully integrated model. We may have seen associations between relevance with past month engagement, if we had a more complete measurement of the model, or other factors such as barriers to use. Second, although the Latino/a/x adult focus is rare in digital mental health research, the sample size was relatively small in general but especially in the follow-up timepoint; the latter could potentially help explain null findings for the model predicting total use over the previous month. The size of the follow-up sample also restricted the number of factors that were explored as determinants of website engagement. Additionally, resource uptake was measured dichotomously, which could underestimate engagement with a single resource (eg, frequency of use). Still, the focus on the Latino/a/x subsample of the larger T4W/Juntos evaluation sample was intentional to describe how this minoritized population engages with digital mental health websites for the purpose of informing future studies. Furthermore, the website was curated for and evaluated with Latino/a/x adults living in California, which should be considered when generalizing findings. Finally, we characterized Latino/a/x adult perspectives generally, but we understand that this ethnic group is not monolithic. Although we did not collect more nuanced demographic data, future research should make efforts to disaggregate by nationality, other languages including indigenous languages (eg, Mixteco), and acculturation to further unpack within-group variation. Indeed, in the second evaluation of the T4W/Juntos website, which is currently underway, we will assess variation by indigenous identity.

### Conclusions

This study helps fill gaps regarding current understanding of Latino/a/x adults’ user experience with public mental health prevention-oriented websites [53]. As challenges with uptake limit the population health impact of digital mental health tools, focusing on usability, comfort, and engagement in Latino/a/x end users is essential to ensure that disparities in access to supports do not widen. Older and Spanish-speaking Latino/a/x adults stand to benefit from targeted support in helping navigate websites and potentially increase their digital literacy. Still, co-design of websites with organizations knowledgeable of end users may promote perceived relevance and user engagement. Aligned with the Behavioral Model for Vulnerable Populations, the severity of behavioral health needs may be a crucial determinant of not only perceived relevance of mental health sites but also actual uptake. Results will act as a pilot for future research on promoting the uptake of digital mental health tools with Latino/a/x communities. Logistically, developers and implementers of digital resources must assess the extent to which end users have access to the necessary technology to engage with such resources. Findings may provide launching points for other investigations of digital mental health tools with diverse samples using this integrated framework that expands the TAM.

## Abbreviations

GAD-2: Generalized Anxiety Disorder 2-item scale
OR: odds ratio
PHQ-2: Patient Health Questionnaire-2
TAM: Technology Acceptance Model
